# Gender and intimate partner violence considerations for cash transfer programming in South Sudan

**DOI:** 10.1186/s41018-025-00181-0

**Published:** 2025-12-03

**Authors:** Allison Jeffery, Angelina G. Akol, Kate Mieth, Morjan Robert Kenyi Duku, Vanessa Saraiva, Shannon Doocy, Gerbrand Alkema, Kevin Savage, W. Courtland Robinson

**Affiliations:** 1 Johns Hopkins Bloomberg School of Public Health, Baltimore, MD USA; 2 World Vision South Sudan, Juba, South Sudan; 3 World Vision United Kingdom, Milton Keynes, UK; 4 World Vision International, Geneva, Switzerland

## Abstract

As the use of cash and voucher assistance increases in South Sudan, there is a continued need to understand its potential interactions with gender norms and intimate partner violence. This paper is part of a larger, mixed methods study whose aims were to measure the association between cash transfer program participation and intimate partner violence (IPV) in Gogrial West, South Sudan; to understand how receipt of cash transfers and the way they are delivered may affect gender relations, power dynamics, and IPV in receiving households, and to identify ways to improve design of cash transfers to mitigate IPV risk and enhance positive effects on gender relations. This paper presents results from qualitative research to investigate gender norms and intimate partner violence in the context of cash programming in Gogrial West, South Sudan. Forty-one key informant interviews, 12 focus group discussions, and 30 individual in-depth interviews were conducted over two rounds of data collection between October 2019 and December 2021. The most common gender norms and IPV behaviors included those relating to livelihoods and unmet needs, household decision-making and household stress, family structures, marital disagreements, and perceived acceptability of IPV. Qualitative analysis suggests that the cash transfer program did not particularly disrupt gender dynamics within households, a situation that would heighten risks of intimate partner violence. More participants reported that the cash program decreased intimate partner violence by reducing household stress, but overall evidence on the direct association of this cash program with IPV is limited.

## Introduction

Cash and voucher assistance (CVA) or cash transfer programming has been used extensively to meet multi-sectoral needs throughout South Sudan. There is mixed evidence regarding the effects of cash and voucher programming on gender-based violence (GBV), including intimate partner violence (IPV). To contribute to the evidence base on gender and intimate partner violence (IPV) considerations for CVA programming in South Sudan, this paper describes the qualitative results investigating the relationship between gender norms and household power dynamics and intimate partner violence in the communities participating in World Vision’s BRACE-II Cash for Work program in Gogrial West, South Sudan.

## Background

South Sudan has been affected by a series of complex crises, having suffered nearly four decades of civil war and persistent underdevelopment prior to gaining independence in 2011 (OCHA 2021a). Conflict broke out again in 2013 and, in conjunction with repeated natural disasters such as flooding, led to a countrywide shortage of food and famine in parts of the country by 2017 (Devi 2017; OCHA 2022a).

Furthermore, the COVID-19 pandemic and resulting lockdowns have continued to increase food insecurity, decrease livelihood activities, and have caused an accompanying rise in gender-based violence (GBV) since 2020 (OCHA 2021a, 2022b, a; REACH Initiative and USAID 2021). Tensions around limited food and finances intensified within households, while policies aimed to contain the spread of COVID-19 (such as movement restrictions, school closures, and social distancing) increased the time women and children spent at home with perpetrators, thus increasing the risk of intimate partner violence within the home (OCHA 2021a, 2022b). The closures of women- and girl-friendly spaces, child-friendly spaces, and other protective activities limited the ability of people experiencing abuse to seek support (OCHA 2021a, 2022b). UN Women has called this global trend of increasing GBV due to the pandemic and related restrictions the “Shadow Pandemic”(UN Women 2021).

The humanitarian crisis in South Sudan is often characterized as a “protection crisis” (OCHA 2022b, a). GBV and IPV are widespread in South Sudan, including in the research sites in Gogrial West (Kircher 2013; Oxfam International 2017; International Organization for Migration 2020; OCHA 2022a). Intimate partner violence in South Sudan has been exacerbated by the humanitarian crisis and resulting psychological and economic stress. Some important contextual factors include increasing militarization and use of violence, widespread human rights violations, displacement, impunity within the legal system, and sharp increases in unmet basic needs (Global Women’s Institute of the George Washington University et al. 2017; Wachter et al. 2018; Murphy et al. 2019; International Organization for Migration 2020; Ellsberg et al. 2020, 2021; Khalaf et al. 2022).

However, IPV in South Sudan is mainly driven by systemic gender inequality—including cultural beliefs around the acceptability of violence against women and children (Oxfam International 2017; Wachter et al. 2018; Murphy et al. 2019; International Organization for Migration 2020; Ellsberg et al. 2020). Both conflict and humanitarian aid can challenge and alter traditional gender roles, particularly through changing who provides and manages household finances (Oxfam International 2017; Wachter et al. 2018; OCHA 2022b). The subverting of traditional gender norms and expectations has been shown to be a driver of IPV (Okello and Hovil 2007; Erikson and Rastogi 2015; Wachter et al. 2018). One study conducted with South Sudanese refugees reported that many men felt that humanitarian programs “usurped” traditional gender roles and that the “western values” propagated by the programs increased their wives’ “disobedience” and disrespect. This in turn triggered men to perpetrate IPV to reinforce their power within the household; however, the study also found that fostering open communication and promoting collaboration and trust between couples could reduce violent behavior (Wachter et al. 2018).

The use of cash and voucher assistance (CVA)^1^ has become a critical part of the humanitarian response in South Sudan and particularly within the food security and livelihoods (FSL) cluster (OCHA 2020, 2021b, 2022c). Conflict-affected populations in South Sudan have also expressed an overwhelming preference for cash programming compared to in-kind assistance. A 2019 survey revealed that 90% of people preferred cash to in-kind assistance, citing greater purchasing flexibility and that cash “can be saved and transported more easily during displacement” (Kiaby 2017).

As a relatively new aid delivery mechanism, there is less evidence on CVA than on other mechanisms, especially regarding its effects on protection outcomes. This is particularly relevant in humanitarian contexts, because there is evidence that rates of GBV, including intimate partner violence (IPV) 2, increase during humanitarian emergencies and armed conflicts (Usta et al. 2008; Clark et al. 2010; Peterman et al. 2011; Vu et al. 2014; Wachter et al. 2018; Wirtz et al. 2018; Stark et al. 2020). In fact, IPV is the most prevalent form of GBV worldwide, including in humanitarian settings (Stark et al. 2010, 2013; Stark and Ager 2011; Hossain et al. 2014a, b; Murphy et al. 2016, 2019). In South Sudan, the ongoing conflict has increased rates of GBV (Wachter et al. 2018; Murphy et al. 2019; International Organization for Migration 2020; Ellsberg et al. 2020, 2021; Khalaf et al. 2022); approximately 65% of South Sudanese women and girls “have experienced physical and/or sexual violence in their lifetime” (OCHA 2022b) and approximately 41–75% have experienced intimate partner violence in their lifetime (International Organization for Migration 2020; Ellsberg et al. 2020; World Health Organization 2021).

Poverty and financial instability are known drivers of IPV that are often worsened in humanitarian settings (Annan and Brier 2010; Erikson and Rastogi 2015; Kohli et al. 2015; Rubenstein et al. 2020), including in South Sudan (Global Women’s Institute of the George Washington University et al. 2017; Murphy et al. 2019; International Organization for Migration 2020; Ellsberg et al. 2021). Thus, CVA is theorized to be an effective way to mitigate IPV, as additional cash within the household could alleviate conflict among couples that is either caused or heightened by the stress of financial insecurity and unmet basic needs (Buller et al. 2016, 2018). However, evidence of CVA’s direct impacts on IPV is mixed—demonstrating positive, negative, and neutral results in both development (Bastagli et al. 2016; Hidrobo et al. 2016) and humanitarian settings (Bell 2015; Buller et al. 2016; Cross et al. 2018; Women’s Refugee Commission and International Rescue Committee 2019; Global Protection Cluster Task Team on Cash for Protection 2020).

A key underlying question is whether and how CVA interventions may impact household dynamics—including how potentially challenging or upending traditional gender norms around livelihoods and household finances could affect or even increase household violence (Bell 2015; Buller et al. 2016, 2018; Cross et al. 2018). One study conducted with South Sudanese refugees suggests that this “usurping” of traditional roles could lead husbands to perceive their wives as being increasingly “less respectful and more disobedient” and lead to increased violence within the home (Wachter et al. 2018). While this finding may not be broadly applicable to all South Sudanese communities, it does highlight the need for protection risk mitigation plans to anticipate and address the ways, which humanitarian interventions, including CVA programs, may challenge traditional gender roles and mitigate any potentially negative consequences (UNHCR 2015; Bell 2015).

Building the evidence base on the effects of CVA on the prevention, mitigation, and response to intimate partner violence is crucial for implementing successful humanitarian programs and policies (Bastagli et al. 2016; Hossain and McAlpine 2017; Cross et al. 2018; Global Protection Cluster Task Team on Cash for Protection 2020). In line with these priorities, World Vision International-South Sudan, with support from World Vision-UK and in partnership with the Johns Hopkins Bloomberg School of Public Health Center for Humanitarian Health, conducted a mixed-methods study of CVA programming in Gogrial West County, Warrap State 3, between 2019 and 2021. Qualitative results are presented in this paper, while a separate paper (under review) presents the quantitative results. The study aimed to answer:

- What is the association between cash transfer program participation and intimate partner violence (IPV) in Gogrial West, South Sudan
- How have the program’s cash transfers affected gender relations, power dynamics, and IPV in receiving households, and
- How might CVA programming in this context be improved in the future to mitigate IPV risks and enhance positive effects on gender relations?

## Methods

The study used a prospective cohort design with participants in World Vision’s Building Resilience through Asset Creation and Enhancement, Phase II (BRACE II) program in six bomas^4^ in Gogrial West. The program provided cash transfers conditional upon household participation in initiatives intended to strengthen food security, nutrition, and resilience. Targeted food-insecure households were provided cash transfers and participated in activities to build or rehabilitate household and community assets such as kitchen gardens, dikes, and roads. The program was delivered across the communities in cohorts staggered over time, providing the cohorts of study. For 8 months, participating households received a cash transfer equivalent to 50% of average household food needs.

Round 1 qualitative interviews were conducted in October–November 2019, around the same time as baseline quantitative data were collected for the first and second cohorts and the control group households. Round 2 qualitative interviews took place in November–December 2021.

For both rounds of the qualitative data collection, semi-structured interview guides were developed in consultation with local community stakeholders. Multi-day qualitative research training, including ethical research conduct, was provided by the study team members from Johns Hopkins Bloomberg School of Public Health and World Vision-South Sudan. For the Round 1 (2019) data collection, training was conducted onsite in Kuajok, Gogrial West; due to COVID-19-related travel restrictions, round 2 (2021) data collection training was conducted using a combination of onsite and virtual activities.

Key informant interviews (KIIs) for both rounds were conducted by trained local interviewers and World Vision staff in Dinka or English. Key informants included government officials, NGO staff, women’s representatives, and other community services professionals. Focus group discussions (FGDs) were conducted in round 1 by a group of trained local interviewers. Due to COVID-19 pandemic-related restrictions on gatherings, individual, in-depth interviews (IDIs) replaced FGDs in round 2 and were conducted by an experienced South Sudanese researcher.

Key informant interview guides included questions about men’s and women’s responsibilities in the household; controls of household finances; what changes, if any, cash transfer programs had on households and communities; if men in the communities ever use violence, against their wives and, if so, under what circumstances; community perceptions of husbands using violence against their wives; and what women in the communities do after their husbands are violent. Focus group discussion guides included five sections: participant demographics, cash transfers (uses and characteristics of recipients), gendered livelihood expectations, household decision-making and power, and intimate partner violence in-depth interview guides included questions about typical marriage practices, decision-making about household finances, and how families respond to difficulties, including disagreements that result in violence.

Interview guides were created and used in English, at the request of the local interview team, who explained that Dinka was not widely used in written form in their communities. Thus, the interviewers verbally translated the questions into Dinka during the discussions, after having completed workshops on standardizing English-Dinka translations. Due to cultural norms precluding the use of recording devices, interviewers took written notes during the sessions. The notes were then typed and sent in English to Johns Hopkins for analysis. A qualitative codebook was designed based on the themes identified during the review of the first round of transcripts. It was then reviewed and approved by the study team. Following this, a single coder coded all the transcripts in Dedoose 8.2.27 software using the approved codebook. Ethical review and approval for the study were provided by the JHSPH Institutional Review Board (IRB) and by the National Bureau of Statistics, Republic of South Sudan.

### Data collection

The first round of qualitative data collection included 22 key informant interviews (KIIs) and 12 focus group discussions (FGDs) conducted in six bomas of Gogrial West, South Sudan (Table 1) between October and November 2019.

**Table 1:** Round 1 respondents

	**FGD** groups (participants)			**KIIs**		**Total by gender**			**Total**
	**Women**	**Men**		**Women**	**Men**	**Women**	**Men**		
Angui	1 (7)	1 (6)		1	5	8	11		19
Lukluk	1 (8)	1 (10)		0	2	8	12		20
Mathiang	1 (6)	1 (7)		5	3	11	10		21
Mayen Gumel	1 (6)	1 (7)		1	0	7	7		14
Monyjoc	1 (6)	1 (6)		2	2	8	8		16
Nyokthiang	1 (10)	1 (8)		0	1	10	9		19
**Total by gender**	**6 (43)**		**6 (44)**	**9**	**13**	**52**	**57**		
**Total**	**12 (87)**			**22**				**109**	

The second round of qualitative data collection included 19 key informant interviews (KIIs) and 30 in-depth interviews (IDIs) conducted in four bomas of Gogrial West, South Sudan (Table 2) in November and December 2021. One key informant interview was excluded due to issues with data quality, leaving 18 for analysis. In-depth interviews with BRACE-II participants and other community members replaced the focus group discussions from the first round due to COVID-19-related restrictions on group meetings.

**Table 2:** Round 2 respondents

	**IDIs**		**KIIs**		**Total by gender**		**Total**
	**Women**	**Men**	**Women**	**Men**	**Women**	**Men**	
Angui	4	3	0	1	4	4	8
Lukluk	6	1	0	1	6	2	8
Mathiang	4	4	1	7	5	11	16
Monyjoc	4	4	4	4	8	8	16
**Total by gender**	**18**	**12**	**5**	**13**	**23**	**25**	
**Total**	**30**		**18**				**48**

The eligibility criteria for rounds 1 and 2 consisted of a combination of both purposive and random selection techniques. The FGD participants (6–10 members per group) in round 1 were randomly selected in each of the six respective Bomas of Gogrial West, with gender disaggregation. The KIIs for both rounds 1 and 2 were purposively selected based on their knowledge of the study objectives including participants from relevant sectors or roles such as protection, Ministry of Agriculture, CVA, and food security and livelihoods (FSL) actors, and community leaders. The in-depth interview participants in round 1 were randomly selected from either the Brace-II participants or the community members.

## Results

### Livelihoods, gender norms, and unmet needs

To understand how the cash-for-work program was situated within the local context, participants were asked about appropriate livelihood activities for men and women. There was agreement among nearly all respondents that women and men have different and rarely overlapping roles. These distinctions were often tied to physical differences between men and women, particularly regarding pregnancy and childbirth.

Respondents agreed that only women should participate in cooking, cleaning, and childcare, as well as small market income-generating activities, such as brewing wine or hosting small tea stands.

The major role of women at home is cooking because in our culture it is mandatory for women to cook for household members even if she gets sick. Any girls from the family should come and cook on that day because if [a man] cooks the whole family and neighbors will no longer respect him for grabbing that role of women and that will lead to disagreement. [Women are responsible for] taking care of children from childhood until they become mature because women are the ones who understand the situation of the child better than men... women are the ones [who] stay home always and because of this our culture states that women have to be responsible [for]… welcoming of visitors and other small things... [Women also cut] grass for constructing houses since construction of houses is divided, men with his side and… she has to cut the grass and if she is not able, she can brew local wine.– KII (F, Round 1), Monyjoc

Strictly defined gender roles also applied to men’s livelihood activities. Men provided the main income streams for their families, often through cattle raising, and were considered leaders of their households. Both men and women referenced men’s physical power and bravery when describing why certain activities were only appropriate for them.

Husbands are responsible for doing many things which wives should not, like hunting, house construction, digging wells, sleeping in the cattle camps during the night, looking for the fertile land or places where people need to migrate. Husbands are also [responsible for leading] any ceremony that needs to be conducted traditionally in the community like naming of the newborn child, wedding negotiation between two groups of bride and bridegroom concerning the dowry. Husbands also have knowledge for making the local bed in the house.– KII (M, Round 1), Mathiang

Men clear big gardens for cultivation to get food for his family members because it is hard for a wife to do because women are considered weak [and] sometimes become pregnant. Therefore, the advice is [for women] not to do heavy work to protect their babies in their wombs. [Men] sometimes go to the forest and cut poles for house construction which is quite difficult for women to do because she cannot manage to walk for long distance and carry heavy things due to the above reasons. It is husbands’ duties to cut trees and burn charcoal mainly to back up the livelihood/resources at the house [which is] also too hard for women because…it affects their fertility when a woman is in first trimester of their gestation period. Husbands take care of cattle at the cattle camp which is difficult for women to do because it needs a lot of effort since driving of cattle needs running because cattle are always scattered and therefore [it] becomes hard for women to do because running after cattle when driving them can affect pregnant women. This may cause abortion and other delivery complications for them. Husbands are considered stronger than wives, this is due to their physical appearance. In this case, what they can do, wives cannot do, like fighting in front wars. This cannot be managed by wives because they are considered fearful and weak naturally because they shed much blood when giving birth to a child. KII (F, Round 1), Monyjoc

In addition to strictly delineated gendered work activities, most livelihood activities available in the community were also classified as inadequate to meet basic needs, leaving much of the community in need of livelihood support. The BRACE-II program was designed to address high levels of unmet need within the community, particularly within the context of the humanitarian crisis. Participants were clear about urgent basic needs in the community, including solutions to flooding, water access via new additional bore holes, money for basic food and non-food items, and ox ploughs for more efficient farming.

The major challenges are the inter-communal clashes, caused by revenge killing, payment of high dowries, thus one has to raid to get the means of marrying, movement of cattle in search of water and greener pasture, political influence etc. where atrocities like rape of women and young girls happen. Cattle raiding affects older women, young girls, old men, and children, this is because elderly people and young children particularly the ones who just stopped breastfeeding are taken to the cattle camp for milk, and young girls are there to take care of these vulnerable groups. Climatic conditions are also contributing factors to the conflict caused by the movement of the cattle in search of grazing land, this is due to the prolonged dry spell.– Key Informant (M, Round 2), Mathiang

### Household financial decision-making and disagreements

The cash transfers provided by the BRACE-II program were unrestricted, so beneficiaries were able to spend the money on anything. Some interview questions focused on how they decided to use the cash to meet their most pressing needs and how household financial decisions were typically made in their communities. Of particular interest was how gender norms affected household financial decision-making, and how increases in household income from the program might impact intimate partner violence.

Questions about financial decision-making and gender produced mixed results during the first round. There was almost an even split between respondents citing men and women as the primary financial decision-makers, with respondents from both groups more often citing their own gender as the decision-maker. Overall, even when there was disagreement about who was the primary decision-maker, there was strong agreement that men made overall financial decisions but delegated to women the role of making day-to-day decisions about household expenditures such as food and schooling.

Women are the ones keeping money and then men direct her on what do and how to use that money, because a man is a responsible person, and if a woman makes her decision to use money or any other resources available at home, a man will feel that his wife disrespects him and that will lead to disagreement.– KII (F, Round 1), Monyjoc

[The] wife is in charge of food items while [the] husband is responsible for the assets like cows, goats, and sheep.– IDI (M, Round 2), Lukluk

[The] man is generally the head of the household [and] thus makes decisions on finances too, but there are incidences where both [men and women] sit and weigh their options on finance. Typically men would use the money on buying livestock, but now some are investing in school fees, diversifying food nutrition, paying for health costs, there have been [many] changes in the financial decision making for the beneficiaries,… [before cash programs,] were the ones that generated income so he just did as he wished but now that women are getting the cash, they tend to consult the man on what they can do with the money, which might not be the case for the non-project participants.– Key Informant (M, Round 2), Mathiang

Most participants also said that men could use violence against their wives when they disagreed with financial decisions or if they determined that their wives made a perceived error in resource use.

Violence begins when the wife misuses household resources. For example, a husband can budget and give money to his wife that can be consumed for one month, if this finishes before the mentioned time, automatically it can cause a beating.– KII (F, Round 1), Mathiang

BRACE-II was noted as the only cash-transfer program in the area. Some participants identified World Vision as the implementing organization, while others did not know which organization was implementing the program.

### Family composition and household resource allocation

Understanding how typical family size, structure, and relationships affect household dynamics—including resource allocation—was also useful for contextualizing norms around intimate partner violence. In this study, almost all participants discussed an ideal family size between seven and eleven individuals, with a husband, wife, and five to nine children. Some also discussed living with in-laws or husbands’ other family members. When asked about family structure in general, co-wives were not often discussed. However, their influence was clear when respondents were asked directly about polygamy in households.

Almost all participants reported that a husband with multiple wives gave the money he earns to his first wife to divide among the rest of the wives. The first wife was expected to consider the number of children in each wife’s household, as well as the newest wife’s responsibility for feeding her in-laws, when distributing assets.

[For] the multiple wives, money could be divided equally starting from the first wife, the second and up to the last. They also consider wives with more children [should] be given more than the rest of the wives but always [the] first wife is given the first chance.– Individual Interview (F, Round 2), Mathiang

Participants discussed a mix of positive and negative effects of polygamy on the co-wives, stating they were both sources of support and advice, and competition for resources and spousal attention. A few interviews mentioned the influence of polygamy and co-wives on intimate partner violence.

Marriage of a co-wife causes a conflict of interest between husband and wife because some women do not like their husband to have more than five wives. Lack of food due to these circumstances, women start to hate their husbands because things are not going well like before, and it leads to divorce.– FGD (F, Round 1), Nyokthiang

Many wives help themselves [by giving each other] advice but [a] single wife has no one advising her on how she should live with her husband.– IDI (F, Round 2), Angui

Respondents suggested that the lack of resources caused or worsened violence. Many referenced intermediary effects between unmet needs and violence, including hunger and stress over meeting basic needs.

Women should be given some work to keep them busy and get income to decrease violence because some violence is caused by economic [causes] which is lack of food and shelters.– KII (F, Round 1), Mathiang

At the household level when there is inadequate of food and there is no hope for food assistance either from organization or relatives…this may cause stress between couples, especially when children are in need of food and husbands have nowhere to get it from. This may let women throw bad words on her husband, and because of stress, the man will not have the heart to control all these insulting statements and this will lead couples to fight.– FGD (M, Round 1), Mayen Gumel

Respondents discussed using cash transfers to meet some basic needs, such as purchasing food, but also mentioned that the cash transfers were not enough to completely meet their household needs. Some participants connected the extra cash to the reduction of stress in the household because meeting basic food needs allowed women to make “mistakes” with food and finances without their husbands reacting poorly. A few participants went on to say that the cash transfers empowered women to become more equal financial decision-makers in their households. One participant more directly tied the cash transfer program to the reduction of violence among beneficiary households.

I am seeing the cash transfer having a lot of positive impact on the community… there is relative peace in the house because the woman does not become a burden again to [the] man asking him for everything and men do not have to go through the quarrelling with the wives for the lack of support.– KII (M, Round 1), Monyjoc

The relationship between couples in a cash transfer program is much improved and less aggressive if compared to non-cash recipient households. This is because one of the major factor[s] contributing to lack of understanding in a household is lack of food and finance for other needs but through the project’s intervention, chances of being disunited are minimized… Many women reported lifting burden from husband and saying they are now happy, participating women are more valued and they improve their outlook.– KII (M, Round 2), Mathiang

Yes, there have been changes [since the start of the cash transfer program], some men who would beat the wives because of hunger and poverty no longer have a reason, and the food will be cooked for them because with cash the woman will buy food… –KII (M, Round 2), Monyjoc

While most participants described a positive, neutral, or no relationship between cash transfers and IPV, some participants noted that, while cash had some important benefits, it could also fuel conflict between couples. Particularly, control of the cash and spending, as well as the influence of alcohol on IPV (including using the cash to buy alcohol), were mentioned.

I would say the project had some benefits, most people were able to invest in animals, mart, clothes, food, and medical services, [but] some used the money on drinking and settling debts. Some men would try to take control of the money, some women would just give their husband something small, others would refuse to let go of the money and… then there is disagreement which might lead to separation.– IDI (F, Round 2), Monyjoc

The husband heard about the cash distribution and when the women have received the money, the man followed her and took all the money and asked the woman to go back and ask for more …We, with the woman’s consent, open[ed] a police case… the man was arrested. The woman said that her husband never helps her with the children they had and her only support came from the cash transfer program, that through the cash she is able to cater for her children’s needs…– KII (F, Round 2), Monyjoc

The above case also included descriptions of the physical violence this woman experienced from her husband; the authors have removed specific and identifiable descriptions to protect the survivor’s privacy. It was unclear in the quote if the violence was specifically instigated by participation in the cash program, though it was implied that intimate partner violence was a persistent, repeating issue in their household.

A few respondents noted that cash was able to successfully mitigate disagreements and violence only among couples whose primary underlying stressor was household finances and who could already communicate effectively with each other. They noted that cash could not alleviate conflict and violence among couples with underlying problems that were not primarily financial in nature, nor among couples with fewer positive communication skills and limited “understanding” of their spouses.

Households participating [in the cash transfer program] and those not [participating] are alike in one way or the other, if the misunderstanding used to be because [of] poverty, then peace set in, but if it is due to alcohol influence, then it is not solved.–KII (F, Round 2), Monyjoc

Yes there have been changes, some men who would beat the wives because of hunger and poverty no longer have a reason, and the food will be cooked for them because with cash the woman will buy food… but for the men that abuse alcohol, there has not been change in such people.–KII (M, Round 2), Monyjoc

### Non-financial marital disagreements and intimate partner violence

Respondents discussed a variety of additional issues relating to marital disputes, sometimes including violence. The appropriateness of resolving marital disagreements was discussed at three levels: between the couple, with family members, and with community leaders. Most agreed that household disagreements should almost always remain between the couple and only involve family members when the couple cannot come to an agreement themselves. Community members were only involved in the rarest cases when families could not come to an agreement or when nothing else worked to bring the couple to a common understanding.

Couples are not supported in solving problems because no one should interfere in home affairs, and it is hard to interfere in a couple’s issue.– FGD (F, Round 1), Nyokthiang

First you sit with your husband in your private place during quiet time and discuss your grievances without other people hearing what you are discussing. If [this] fails, you can involve family members; either his elder brother, father, friends or neighbors, so that they [can] address the case. Finally, if he still persists, then the last stage is going to court.– KII (F, Round 1), Monyjoc

Participants discussed under what conditions it is considered acceptable for a husband to use violence against his wife. In addition to disagreements regarding perceived misuse of household resources and finances, common responses included when a wife does not complete household tasks, drinks alcohol, refuses sex, or is suspected of adultery.

Yes, husbands beat their wives when wives fail to do her household activities like cooking food for the family and taking good care of her children. Husbands also beat their wives sometimes when wives do things which are not necessarily liked in our culture; women or wives should not be away [from] home [until] late hours, especially at nighttime. If the women or wives do that or drink alcohol while she is still young, it can let the husbands beat or quarrel with their wives.– KII, (M, Round 1), Monyjoc

[In reference to acceptable conditions for husbands to use violence against their wife] When she refuses to have sex with you, because you are entitled to have sex with her at any time you feel [like it] and she must accept it…– KII (M, Round 1), Angui

Adultery and suspicion of adultery were frequently mentioned in the first round of data collection as common reasons men committed violence against their wives. Round 2 interviews then asked specific questions about the perception of, frequency of, and response to adultery. All respondents said that adultery is a crime. Additionally, despite adultery being mentioned frequently in interviews, all respondents said that it almost never happens in their villages and is only prominent in towns and cities. Many participants also implied that only women could technically be adulterers in the criminal sense and that men would only be considered cheaters for the same acts. Of note, in South Sudan, it is common for survivors of rape to be labeled adulterers (Government of South Sudan et al. 2020; Ellsberg et al. 2020, 2021).

The community considered adultery or cheating as [a] criminal act. But [men cheat often, while] adultery cases are minimal. An adulterer or cheating person is acknowledged as a sinful person in the society…[adultery] is not common in this community but [it is] in the towns, this is how we hear about adultery cases.– IDI (M, Round 2), Mathiang

Many respondents referenced dowries, or that men had purchased women, and they were therefore, the men’s property, as justification for their violence. Others mentioned dowries as a reason to respect women’s choices around sexual relations, in certain circumstances.

[Men use violence] when a woman committed adultery - because we pay many cows in [the] form of dowry to marry them and therefore, we expected them to be loyal to one man and respect him no matter what may happen to the man - in case there are weaknesses from the man^5^, she can wait until her husband authorizes her to look [for] another man within family.– FGD (M, Round 1), Lukluk

The high bride price is one of the factors that lead to men mistreating women; they expect these women to be perfect, [and that they] should not mismanage the resources…The woman should be working like servants and are expected to bear children, if not then it is a big deal. Most of the disagreements lead to physical violence, some [women] die and others run off to their parents and [then] she is talked to and asked to submit to the man since he married her, [and she] should not be at loggerheads with him, so the man gets the upper hand and the woman is returned home.– KII (F, Round 2), Monyjoc

A woman can deny sex from her husband. This happens when she is [tired] or sick, therefore you can respect her opinion and give her time to rest or get recovered from her sickness because what matters is understanding and compromise between couples. Since we pay 100–300 cows [for a woman] if you want to be respected in the community.– Focus Group Participant (M, Round 1), Nyokthiang

### Community perceptions of and responses to intimate partner violence

Respondents had mixed opinions on community perceptions of IPV. Some reported that IPV would lead perpetrators to be labeled as “bad husbands,” while others stated that the community often finds IPV justifiable. Other respondents discussed intimate partner violence as necessary for a wife to respect, understand, or obey her husband.

People in the community think that the husbands who use violence against their wives are not good husbands and the people used to think to tell them some advice to not [use] violence against their wives. People also think or tell the husbands that, instead of using violence against the wives, [it] is better to leave or divorce her.– KII (M, Round 1), Mathiang

And if the husband beats the wife badly in the community, he will be first investigated by the community members to find out the actual reason for beating his wife and then later judged innocent or guilty. Sometimes women behave like children so they can be treated like children regarding their rights.– KII (F, Round 1), Monyjoc

Women are always tolerant and merciful as a gift to them [from] God and as such they tolerate and forgive stresses [and violence] and continue with their home activities and hope for change… Others feel at home or comfortable after they are beaten or tortured and then start to respect their men and do the home activities/work [efficiently].– KII (F, Round 1), Monyjoc

Most men still use both verbal and physical violence against their wives even today. The community believes that it is the only way to get a woman to obey the man, they use verbal abuse to intimidate women, one would call the woman useless, dirty and worthless trash he picked and improved.– KII (F, Round 2), Mathiang

A minority of respondents described intimate partner violence as unnecessary and even fewer described it as unacceptable. In contrast, when directly asked if anything about men’s treatment of women should change, more people reported that men should avoid using violence with their wives than had noted that violence was unacceptable. This could be influenced by social acceptability bias, in that the first question asks for personal opinions on the acceptability of violence and the second asks for a summary of community attitudes. There were also a few respondents, particularly women, who said it was not their place to recommend how men should act in their own households.

Most men are doing their best to treat their wives well, some even accompany their wives to the hospital when they are about to deliver [a baby], they also make sure to provide them with nutritious food when they are pregnant, foods like fish and greens. Yes, for the few men that beat their wives, I think they need to know that it is not okay and need to resolve differences through dialogue.–IDI (M, Round 2), Mathiang

I do not have any right to suggest how a man should treat his wife or behave in his own house, honestly it is upon the man and how he runs his house. Beating of women is bad yes, but some women are beaten because they do not want to listen and submit, and [for] others it is due to alcohol, so I believe the change on individuals drinking alcohol and beating their wives is needed. Also, young women who [drink] alcohol should be advised to stop, some actually neglect their young children to hunger and diseases thus they die, so women have to also change.– IDI (F, Round 2), Angui

Participants discussed interpersonal and community responses to intimate partner violence, including the adequacy of the responses. Many agreed that violence is an internal family matter and that a woman should take care not to embarrass her husband publicly. In some situations, as with other marital disagreements, it was considered appropriate to involve the family and community leaders when couples cannot resolve a severe disagreement amongst themselves.

What the women would do after the husband [was] violent with her, she [would] go back home to her parents according to our culture and then her parents would call the parents of her husband to come and sit together to find out who is wrong and who is right between husband and women.– KII (M, Round 1), Mathiang

Elders and neighbors should intervene immediately whenever… couples disagree to stop them from arguing or fighting. Forgiveness matters a lot since there is a love between the couples [that] may encourage them to forgive each other in the house. If the case is beyond the capacity of the elders and relatives, then the case should be forwarded to the concerned authorities like police and judicial courts.– KII (F, Round 1), Monyjoc

Some participants specifically described responses to rape within the community, including exclusion of women who have been raped from community life and punishment for perpetrators. Some women who have experienced rape were forced to marry their perpetrators, and in other situations, the woman’s family would seek revenge and attack the perpetrator or a member of his family.

Raped girls or women have less respect from the family members as well as from neighbors and she cannot be allowed to participate in sharing ideas at home since she had committed [a] crime, though it was not her will. That is why some women don’t want to report [being raped] since [it] turns to shame.– FGD (M, Round 1), Mayen Gumel

Some women are abused for their daughter’s mistakes like getting pregnant or being raped, then the mother is blamed and insulted, she in turn blames the girl for being raped which is entirely not their fault…There has been awareness of GBV, but the community is still very rigid to change on the use of violence all because they believe that if the woman is not beaten she would never listen.– KII (F, Round 2), Mathiang

[A woman who is raped should] report it to local authorities available in the area, for example the sultan [chief] and elder groups to handle it according to traditional norms… While if it is a daughter, eventually her brothers and relatives have to mobilize to go and attack the person who raped their daughter because girls are our main sources of income in our society.– FGD (M, Round 1), Mayen Gumel

Women’s options for leaving violent situations were limited, especially as they were often not able to take their children with them if they left their households. Suicide was discussed as a common option for women experiencing sustained violence.

[A] woman may decide to refuse do any her household activities that need to be done by women like cooking, milking cows, and not show respect to her husband. Sometimes women decide to leave [and go] to her [family] house…she may decide to kill herself and…also the woman thinks of committing adultery with another man.– KII (M, Round 1), Mathiang

The only thing that will come to her mind first is to escape in order to be safe/secure, because life is so important… [She] may stay because the husband paid the dowry so there is no way to go and leave the children alone. Sometimes if you escape, he may think of getting married to [a] new co-wife who will later come and mistreat the children badly, so it`s better to stay with your children than [to] leave them to be mistreated.– KII (F, Round 1) Mayen Gumel

Others commit suicide when fighting remains unstoppable at home and for her to be free she has to kill herself by hanging herself with rope or even poisoning herself with hair dye and other chemicals. Many [women] look for reconciliation measures by calling her husband’s relatives, neighbors, colleagues and friends to intervene and solve the problem.– KII (F, Round 1), Monyjoc

Lack of comprehensive services to prevent and respond to IPV was frequently cited as an issue in the participants’ communities. Some participants also suggested changes that would be helpful to preventing and responding to IPV in their communities.

No services are available here for these women [who experience violence] because the government is not bringing these services to us. The services needed but not available are a women and child protection unit, medication, and security, such as a police station [that] should be establish[ed] near the community to maintain the law. Training about GBV and psychological first aid [is] also needed to assist survivors. The assistance of food…also needs to be available.– KII (M, Round 1), Monyjoc

Creating the point of accountability [for those who] start the violence. Empowering women to have equal standing with men and share the same powers. Participation of women in decision making. Avoiding discrimination between the families can end disagreement.– KII (F, Round 1), Mathiang

## Discussion

Worldwide, CVA is generally considered a safe and even protective aid modality, with either few negative effects, no effect, or some positive effects on reducing IPV risk (Bastagli et al. 2016; Cross et al. 2018; Buller et al. 2018; Women’s Refugee Commission and International Rescue Committee 2019). However, in some cases, CVA can increase the risk of intimate partner violence “particularly if women are the direct recipients of assistance and they do not typically control household resources; or if men are marginalized in aid delivery and/or in the wider economy” (OCHA 2015). Given this, contextual gender and violence norms are expected to influence the effects of CVA, with CVA either upsetting, reinforcing, or working within gender norms; each of these options would likely have different effects on intimate-partner violence. It is crucial that humanitarian actors developing CVA programs consider community norms about gender and intimate partner violence to design and implement safer and more effective activities.

### Linking cash and voucher assistance and IPV

Results regarding the direct association between CVA and IPV were limited and mixed. Many participants discussed the inability of families to meet basic needs, which was often cited as directly influencing overall household stress and thus the likelihood of violence against women. Disagreements between spouses about how to manage or spend limited financial resources, as well as husbands’ perception that their wives were “misusing” or “mismanaging” household finances and were both described as direct drivers of IPV.

CVA seemed to most benefit couples whose disagreements were primarily financial in nature and who already communicated clearly. The cash program seemed to have limited ability to mitigate—and in some cases even escalated—conflict between couples whose underlying issues extended beyond household stress due to unmet needs. These underlying non-financial issues tended to relate to known social drivers of IPV, such as women acting outside of established gender norms (such as not fulfilling household tasks or being outside late at night), alcohol use, or adultery and perceived adultery (Global Women’s Institute of the George Washington University et al. 2017; International Organization for Migration 2020; Ellsberg et al. 2020, 2021). They were further justified by the community’s general acceptance of men’s use of violence in these situations.

### Contextualizing CVA within existing gender and IPV norms

The quantitative component of the mixed-methods study indicated that composite rates of IPV (combining psychological, sexual, or physical violence) among female respondents in the 12 months preceding 2021 interviews were 60.5% for participants in the cash transfer program and 65.3% for controls (non-participants in cash programming) (manuscript in review) 6. Actual rates may be higher, as stigma, shame, and lack of awareness of women and girls’ rights, as well as lack of access to services and opportunities for redress, cause GBV and IPV to often go unreported (Ellsberg et al. 2001; Vu et al. 2014; International Organization for Migration 2020).

As noted above, a previous study found that fostering open communication and promoting collaboration and trust between couples could reduce violent behavior (Wachter et al. 2018).

In this study, participants mentioned that engaging in dialogue or open conversation was a potential conflict-resolution method for couples looking to resolve differences. However, a potential barrier to increasing open communication between couples may be the common belief that household matters should remain private between the couple. This finding is consistent with current literature (Erikson and Rastogi 2015; García-Moreno et al. 2015) and may make programming about improving couples’ communication less culturally acceptable.

This study found that patriarchal cultural and gender norms played key roles in most aspects of life, including expectations around acceptable livelihoods and daily tasks, household and community power hierarchies, family structures, financial decision-making, and relationship dynamics—including intimate partner violence.

Men were described as more courageous and physically fit; they were therefore expected to be primary breadwinners and heads of household, conduct more physically demanding tasks, make overarching financial decisions, and take on community leadership roles. They were also seen as having the right to control and discipline their wives and children as they saw fit.

Women were often described as fragile, partly due to pregnancy and childbearing. They were expected to manage household activities such as child-rearing, cooking, cleaning, and day-to-day financial decision-making. Cultural norms suggested women should be subservient to their husbands. These norms were reinforced by the belief that men had “paid” for their wives via dowries and that they were thus property. Women were often subjected “discipline,” including violence, by their husbands; this was reported to primarily occur when husbands perceived their wives as (1) mishandling daily tasks such as mismanaging daily finances and not keeping up with housework, (2) challenging the power hierarchy within the household such as refusing sex and disagreeing with husbands’ decisions), or (3) participating in culturally deviant behaviors including adultery, staying out of the house late at night, and consuming alcohol. Such behaviors were also often perceived as a wife actively disrespecting her husband.

Only a few respondents described violence against women as unnecessary or unacceptable. However, when participants were later asked if men’s treatment of women should change, more people reported that men should avoid using violence with their wives than had originally reported that violence was unacceptable. This presents an opportunity to build on positive deviance towards more gender equitable practices.

Because women were largely in charge of day-to-day finances, they were more vulnerable to intimate partner violence as “punishment” if their husbands disapproved of how they spent scarce household resources. Additionally, women were generally confined to a very limited set of appropriate livelihood activities outside of homemaking, which likely restricted families’ access to potential additional sources of regular income outside of the BRACE-II program.

IPV might be expected to increase from baseline levels when the program disrupts strict gender norms in communities with already heightened acceptability of intimate partner violence. This can be demonstrated in situations where women gain financial power (and therefore household power) from CVA, and men use violence to regain their dominant household position (Nuwakora 2014 via Buller et al. 2018; Bobonis et al. 2013 via Bastagli et al. 2016). As this study demonstrates, these communities in Gogrial West generally have strict gendered roles and expectations, patriarchal power structures including the dowry system, and high acceptability of violence against women. Given these contextual factors, one could expect that, if the BRACE-II cash transfer program upended gender and power dynamics within participating households, men’s perpetration of IPV would be expected to increase in attempts to regain their position of power.

However, the qualitative component of this study found mixed results, with most participants not connecting the transfers to IPV at all. Of those who did connect the transfers to IPV, most expressed that the transfers caused a decrease in IPV perpetration through easing household economic stress. This may be because the cash amount was not sufficient to upend household gender dynamics, as the transfer funds were within the amount women were already expected to use for day-to-day household spending, and they generally bought the things their husbands expected them to purchase.

Had the cash amounts been larger and therefore considered a more strategic financial asset, there may have been a different impact on IPV. While more completely meeting household needs could further reduce household stress and therefore decrease intimate partner violence, giving women access to significant strategic assets could have alternatively increased IPV by upending men’s household financial control in communities with rigid gender norms. In Gogrial West, cattle were often seen as the strategic financial assets and were firmly within men’s responsibility and control; thus, it is unclear if providing women beneficiaries with cattle instead of cash would have more significantly upended power dynamics and increased violence.

Integrating CVA programs and protection services, including mental health and psychosocial support for individuals and couples, as well as case management and referrals for those at high risk of or experiencing IPV, is important to prevent, mitigate, and respond to IPV. These could be especially effective if coordinated with the humanitarian actors already conducting GBV prevention and response activities, including direct service provision and capacity strengthening of local services (Government of South Sudan et al. 2020).

### Limitations

This study had various limitations due to contextual factors and data collection methods, as well as outside factors such as COVID-19.

#### Contextual factors and data collection methods

Due to local community sensitivities about the use of recording devices, none of the interviews was recorded. Instead, the bilingual interviewers (fluent in English and Dinka) asked questions in Dinka but took notes on the respondent’s comments in English, as written Dinka was not commonly used nor standardized in these communities. KIIs and IDIs were conducted by a single interviewer, while FGDs were conducted by two enumerators—one facilitating the discussion and the other taking notes. Given the lack of verbatim transcripts and the need to do on-the-spot translations from Dinka to English, it is possible that the original meaning was altered in translation, or the comments were not recorded accurately.

The two rounds of interviews were conducted several years apart (late 2019 and late 2021) by different interviewers in each round and with mostly different respondents. Data collectors sometimes reported a lack of privacy due to a lack of nearby structures on the feeder roads where interviews were taking place. They additionally reported somewhat inconvenient timing of interviews, as groups of beneficiaries often walked home together, and thus had to wait for the interviewee to complete the interview process, presumably putting additional time pressure on the interview. Both factors could have limited full open participation in the study. The lead data collector also noted that interviewing community leaders could have biased some information about intimate partner violence, as local leaders often protected, rather than punished, perpetrators in their communities.

#### Outside factors and COVID-19

The COVID-19 pandemic began in the middle of this research study. While the study team was able to add additional questions regarding COVID-19’s impact during the round 2 data collection, respondents described little to no direct effect on daily life in their communities, as COVID-19 was perceived as a disease that was mostly spread in larger towns and cities.

However, some participants mentioned the indirect effects of the COVID-19 pandemic and its related policy measures, including lockdowns and social distancing measures, as having some negative effect on daily life. A few participants mentioned that the pandemic was negatively affecting the availability of jobs, physically separating families and community members from each other, and making the future generally unpredictable, which was negatively impacting the communities’ ability to develop and implement longer-term plans. All these factors may have impacted interviewees’ answers between the first and second rounds of data collection and may have been additional risk factors for IPV perpetration.

## Conclusions and recommendations

Overall, while the results of the mixed-methods study demonstrate evidence of IPV among households in Gogrial West, there was no clear unidirectional association between participation in the BRACE-II program and experience of IPV. In the quantitative study (manuscript in review), the association was not statistically significant, while in the qualitative study, some respondents suggested that cash transfer program participation was protective from IPV and others reported increased risk. Therefore, while a link may exist, this study cannot draw broader conclusions on the impact of cash transfers on IPV. However, the qualitative findings do suggest that the BRACE-II CVA program likely did not disrupt gender dynamics in a significant enough way to create a clear demonstrable positive or negative effect on intimate partner violence. The specific BRACE-II program components, including cash transfer amount and local contextual norms and conditions, limit study findings to the context of Gogrial West; findings may be different in other parts of South Sudan and in other humanitarian contexts.

With these caveats and limitations, and understanding that humanitarian actors will likely increase the use of CVA in South Sudan, these results support the importance of understanding the potential positive, neutral, and negative consequences on IPV in specific affected communities, before, during, and after CVA program design and implementation. This study offers some recommendations applicable across various contexts, which align with key CVA and protection guidance (UNHCR 2015).

### Conduct a gender analysis, with a focus on household dynamics and IPV, to understand community perceptions of IPV

Gender analyses should focus on the distribution of power within the communities, including around livelihoods, family structure, and other gendered expectations and norms. Women’s autonomy and empowerment, especially around financial power and decision-making, are particularly important. It is also crucial to understand norms about violence, as well as existing prevention and response services. If this analysis has not already been conducted by another actor, mapping the organizations working in these areas is critical to improve contextual knowledge and to establish referral pathways for protection services. If analysis finds that strict gender norms are common, it is important not to overcorrect by opting to only give cash to men to avoid risks, but to instead create a gender action plan with a comprehensive risk mitigation plan and integrated protection referral program, supported by a robust monitoring system that would identify changes in household dynamics, whether positive or negative.

### Plan for a robust protection program, especially if the gender analysis finds that CVA programs will significantly challenge power systems and structures

Protection programs are life-saving interventions in humanitarian emergencies that prevent, mitigate, and respond to risks of violence, abuse, and exploitation. They are even more important if humanitarian CVA programs are likely to challenge power systems and structures, even if CVA is not being implemented with the explicit intention to change gender norms or to decrease IPV. Programs should include a safe and accessible reporting mechanism, comprehensive case management and/or referrals, women’s support groups, and women’s participation on project oversight committees. When community basic needs have been met, behavior change activities promoting gender equality and positive masculinities should also be considered to support sustained IPV and GBV reduction.

### Conduct additional research on CVA and violence

CVA programs with different cash amounts, transfer periods, and reliability may have different effects on IPV. In this case, because the community was largely experiencing acute food insecurity at IPC 3–4^7^ (levels which necessitate humanitarian intervention), the transfers combined with existing resources were not perceived as sufficient to meet basic needs. Further research is needed to determine if a program with larger transfer amounts that met basic needs would have different effects on IPV (USAID 2022).

Further study is also needed on the intersection between intimate partner violence and violence against children. There is a known connection between violence against women (VAW) and violence against children (VAC), as they often occur within the same households, share risk factors (including gender inequality, marital conflict, lack of responsive institutions, and legal impunity), and can have “cumulative, compounding effects” on physical and mental health outcomes within the household (Guedes and Gennari 2021). Moreover, a recent literature review conducted by Save The Children concluded that CVA “largely promotes child protection [outcomes], particularly for outcomes that are directly influenced by financial barriers or burdens” (Salloum 2021), citing results from a 2017 synthesis brief by Mishra and Battistin (Mishra and Battistin 2017). This study did not include specific questions regarding violence against children, limiting our ability to examine violence in a holistic way, which is becoming increasingly critical for maximizing impact within the protection sector. Future studies should explore the links between VAC, VAW, and cash in humanitarian settings, especially considering the expressed need for programs to concurrently address the intersections between VAW (including IPV), VAC, and cash-based programming (Guedes and Gennari 2021).

There are still clear evidence gaps regarding how different design and implementation approaches of CVA programs might interact with existing gender norms to impact IPV prevalence in humanitarian settings. While there is evidence on the potential of CVA interventions to positively affect gender equality and women’s empowerment, it is often contingent on the CVA being paired with complementary activities such as social programs and skills training (Bell 2015; Buller et al. 2016, 2018; Cross et al. 2018; World Food Programme 2019). Thus, this study concurs with previous findings (Bastagli et al. 2016; Cross et al. 2018), that to design the most effective CVA program while mitigating potential increases in IPV or even designing a “CVA for protection” IPV reduction program, it is crucial to specifically assess community contextual norms related to gender and intimate partner violence, as well as ensure integrated protection activities and referrals.

## Data Availability

The datasets used during the current study are available from the corresponding author on reasonable request.

## Abbreviations

BRACE: Building Resilience through Asset Creation and Enhancement
CVA: Cash and voucher assistance
FGD: Focus group discussion
FSL: Food security and livelihoods
GBV: Gender-based violence
IASC: Interagency Standing Committee
IDI: In-depth interview
IPV: Intimate partner violence
KII: Key informant interview
USD: United States dollars
VAW: Violence against women
VAC: Violence against children

## Footnotes

1 The Cash Learning Partnership (CALP) defines cash and voucher assistance (CVA) as “the direct provision of cash transfers and/or vouchers for goods or services to individuals, households, or group/community recipients. In the context of humanitarian response, CVA excludes payments to governments or other state actors, remittances, service provider stipends, microfinance, and other forms of savings and loans” (The CALP Network 2023).

2 The WHO defines IPV as “any behavior within an intimate relationship that causes physical, psychological, or sexual harm to those in the relationship” and includes physical violence, sexual violence, emotional (psychological) violence, and controlling behaviors, such as “isolating a person from family and friends; monitoring their movements; and restricting access to financial resources, employment, education, or medical care” (World Health Organization 2012).

3 From 2011 to 2015, Gogrial West County was in Warrap State. Between 2015 and 2020 (both when South Sudan had 28 and 32 states), Gogrial West County was in Gogrial State. On February 22, 2020, a transitional coalition government formed, and the number of states returned to ten, with Gogrial West County in Warrap State.

4 Bomas are the lowest level administrative unit, in South Sudan, below payams and similar to a village, block or ward in other contexts. Bomas vary in size and many contain multiple, smaller villages.

5 “Weaknesses from the man” traditionally refers to situations where a man is unable to have sexual relations or is otherwise unable to have children. In these cases, some men choose to send their wife or wives to other male family members, as the whole family has paid the dowry.

6 Although the composite IPV rates were slightly lower among respondents participating in the cash transfer program as compared with non-participants, these differences were not statistically significant, leading the authors to conclude that, although IPV was present in both study groups, participation in a cash transfer program did not have any significant association, positive or negative, with experiences of IPV.

7 IPC is the Integrated Food Security Classification scale, ranging from IPC 1 (“minimal”) to IPC 5 (“famine”). IPC 3 is considered “crisis” level, and IPC 4 is considered “emergency” level. (USAID 2022).
